# Optimization of an Extraction Solvent for Angiotensin-Converting Enzyme Inhibitors from *Hibiscus sabdariffa* L. Based on Its UPLC-MS/MS Metabolic Profiling

**DOI:** 10.3390/molecules25102307

**Published:** 2020-05-14

**Authors:** Mohamed A. Salem, Haidy E. Michel, Marwa I. Ezzat, Mona M. Okba, Ahmed M. EL-Desoky, Shanaz O. Mohamed, Shahira M. Ezzat

**Affiliations:** 1 Department of Pharmacognosy, Faculty of Pharmacy, Menoufia University, Gamal Abd El Nasr st., Shibin Elkom 32511, Egypt; mohamed.salem@pharma.cu.edu.eg; 2 Department of Pharmacology and Toxicology, Faculty of Pharmacy, Ain-Shams University, Cairo 11566, Egypt; heidieffat@pharma.asu.edu.eg; 3 Pharmacognosy Department, Faculty of Pharmacy, Cairo University, Kasr El-Ainy Street, Cairo 11562, Egypt; marwa.ezzat@pharma.cu.edu.eg (M.I.E.); mona.morad@pharma.cu.edu.eg (M.M.O.); 4 Department of Molecular Biology, Genetic Engineering and Biotechnology Research Institute (GEBRI), University of Sadat City (USC), Sadat City 32958, Egypt; ahmed.desoky@mynaturalwellness.com; 5 School of Pharmaceutical Sciences, Universiti Sains Malaysia, Penang 11800, Malaysia; shanaz.mohamed@mynaturalwellness.com; 6 Department of Pharmacognosy, Faculty of Pharmacy, October University for Modern Sciences and Arts (MSA), Giza 12451, Egypt

**Keywords:** ACE inhibition, *Hibiscus*, metabolomics, LC/MS, chemometry, extraction

## Abstract

*Hibiscus* species (Malvaceae) have been long used as an antihypertensive folk remedy. The aim of our study was to specify the optimum solvent for extraction of the angiotensin-converting enzyme inhibiting (ACEI) constituents from *Hibiscus sabdariffa* L. The 80% methanol extract (H2) showed the highest ACEI activity, which exceeds that of the standard captopril (IC50 0.01255 ± 0.00343 and 0.210 ± 0.005 µg/mL, respectively). Additionally, in a comprehensive metabolomics approach, an ultra-performance liquid chromatography (UPLC) coupled to the high resolution tandem mass spectrometry (HRMS) method was used to trace the metabolites from each extraction method. Interestingly, our comprehensive analysis showed that the 80% methanol extract was predominated with secondary metabolites from all classes including flavonoids, anthocyanins, phenolic and organic acids. Among the detected metabolites, phenolic acids such as ferulic and chlorogenic acids, organic acids such as citrate derivatives and flavonoids such as kaempferol have been positively correlated to the antihypertensive potential. These results indicates that these compounds may significantly contribute synergistically to the ACE inhibitory activity of the 80% methanol extract.

## 1. Introduction

*Hibiscus sabdariffa* Linn. (HS), Malvaceae (Syn: Roselle, Rozelle, red sorrel, Sour tea and Karkade) has been extensively used in different systems of medicine as antihypertensive tea [1]. HS extracts were also reported to possess potent an in vitro antioxidant effect, which was attributed to the presence flavonols, anthocyanins and tannins [2,3]. Animal studies have shown that consumption of HS extract reduces blood pressure in a dose dependent manner [4,5]. Administration of the HS aqueous extract to salt-induced hypertensive albino rats for 28 days showed a significant reduction in diastolic and systolic blood pressure when compared to the normotensive and hypertensive rats [5]. Another study showed that the crude methanolic extract of HS possessed a vasodilator effect in the isolated aortic rings of hypertensive rats [1].

In addition, the daily consumption of HS tea significantly lowered the systolic blood pressure and diastolic blood pressure in adults with mild to moderate essential hypertension [4,6,7]. Additionally, the aqueous extract of HS showed a dose dependent diuretic and natriuretic that may be mediated by nitric oxide release [8]. HS aqueous extract (150 mg/kg/day for 4 weeks) reduced the serum angiotensin converting enzyme (ACE) and plasma aldosterone in mild to moderate hypertensive Nigerians with equal efficacy as Lisinopril [9]. Moreover, the anthocyanin rich fraction of HS calyces showed ACE inhibition activity [10].

Extraction solvents were reported to have an influence on the nature and the amount of secondary metabolites extracted from medicinal plants. Thus, the choice of the proper extraction solvent is necessary for the desired pharmacological activity of these extracts [11]. Optimization of the extraction method to obtain the most biologically active extract is a very important step that needs great attention. Many studies reported the antihypertensive activity of *Hibiscus*, however the proper solvent for *Hibiscus* extraction has not been specified. Metabolomics, the large scale analysis of the whole set of metabolites within a biological system, has emerged as an indispensable tool for natural products drug research [12].

Hence, the present investigation was undertaken to specify the most active antihypertensive extract of HS through preparation of different extracts of HS dried calyces and determination of their ACE inhibition activity, which was correlated to their chemical profiling using ultra-performance liquid chromatography (UPLC)-MS/MS analysis and chemometric analysis.

## 2. Results

### 2.1. ACE Inhibition Assay

Concerning the ACE inhibition activity (Table 1), H2: 80% methanol extract showed the highest inhibition capacity with IC50 of 0.01255 ± 0.00343 µg/mL, followed by H3: 100% water and H5: 70% ethanol (IC50 0.2058 ± 0.05045 and 0.6390 ± 0.032 µg/mL, respectively). It is worthy to mention that the activity of H2 and H3 exceed that of the standard captopril with IC50 0.210 ± 0.005 µg/mL.

### 2.2. UPLC-MS/MS Analysis and Metabolite Identification

We used ultra-high performance liquid chromatography coupled to high resolution mass spectrometry (UHPLC/HRMS) analysis for the untargeted identification of metabolites from different extraction methods. Peaks from raw chromatograms were aligned by their parent masses, chemical noise was subtracted, and a final alignment file of all chromatograms was the obtained, which contained information about *m*/*z*, retention times (RT) and intensities of each annotated peak. The peaks were assigned using an in-house *Hibiscus* metabolite database, according to the retention time and parent masses. Identification of metabolites was based also on the comparison of acquired mass spectra with previously published data on *Hibiscus* [10,13,14,15,16].

Our analysis revealed the presence of about 36 identified metabolites, including flavonoids, anthocyanins, phenolic and organic acids (Table 2). Identification of these metabolites was also confirmed based on extracted ion chromatograms (EIC) and then comparing the MS and MS/MS data to common MS databases such as METLIN (http://metlin.scripps.edu), MassBank (www.massbank.jp) and the Human Metabolome Database (http://www.hmdb.ca/). An accuracy error of 5 ppm was set in the MS search and the fragments were verified in MS/MS search. Besides, the characteristic molecular ion and corresponding fragments were confirmed to have coelution pattern.

**Figure 1:**
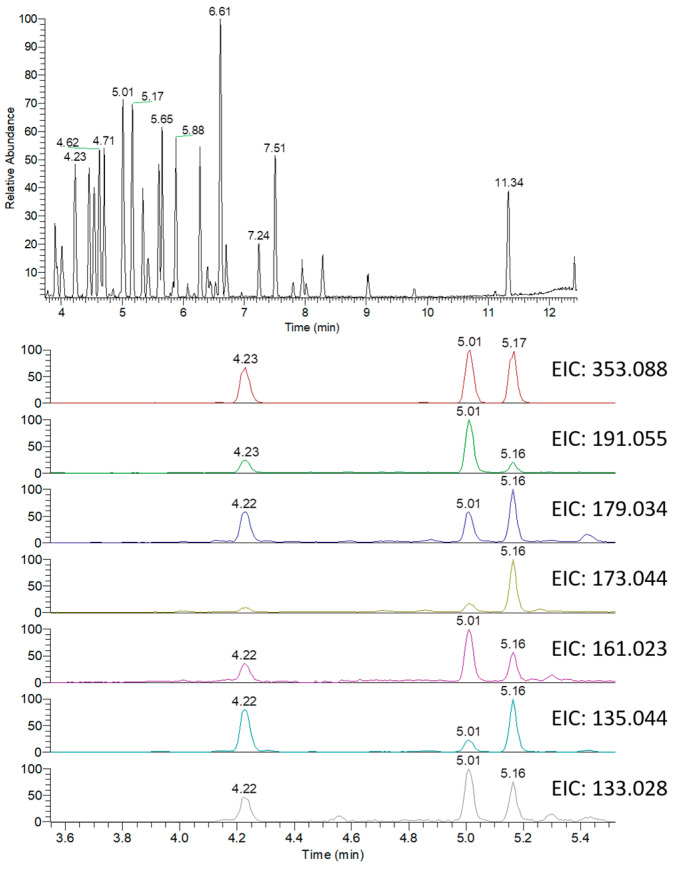
Base peak chromatograms (BPC) and extracted ion chromatograms (EIC) of the peak at *m*/*z* representing caffeoylquinic acid isomers from the *Hibiscus* extract measured in the negative ionization mode.

To illustrate the identification process, an exemplary detail is provided for the identification of caffeoylquinic acid (CQA) isomers. CQA isomers are one kind of the polyphenol family of esters that are most characteristic in *Hibiscus* among other plants [17]. There are four free hydroxyl groups at 1-, 3-, 4- and 5-position of quinic acid that can be esterified with the carboxyl group of caffeic acid. Different CQA isomers have the chemical formula C16H18O9 and the monoisotopic mass of 354.095085. The extracted ion chromatograms (EIC) of the peak at *m*/*z* 353.087809 that represents [M − H]^−^ of caffeoylquinic acid showed three different peaks at RT 4.23, 5.01 and 5.17 min indicating at least three CQA isomers (Figure 1). The characteristic fragment ions of CQA, obtained from MS/MS data of previously published CQA standards [18,19], showed a coeluting pattern with the corresponding molecular ion (Figure 1).

The most characteristic fragments included ester bond cleavage between caffeoyl and quinic acid leading to fragments at *m*/*z* 191.05557 [quinic acid − H]^−^, 179.03427 [caffeoyl − H]^−^, 173.04462 [quinic acid − H − H_2_O]^−^, 161.02359 [caffeoyl − H − H_2_O]^−^, 135.04417 [caffeoyl − H – COO]^−^ and 133.02846 [caffeoyl − H − H_2_O − CO]^−^ (Figure 1). Comparing the relative abundance of fragment ions in MS/MS with the previously published CQA fragmentation pattern [18,19], the three chromatographic peaks at RT 4.23, 5.01 and 5.17 min can be tentatively assigned to their specific CQA.

**Figure 2:**
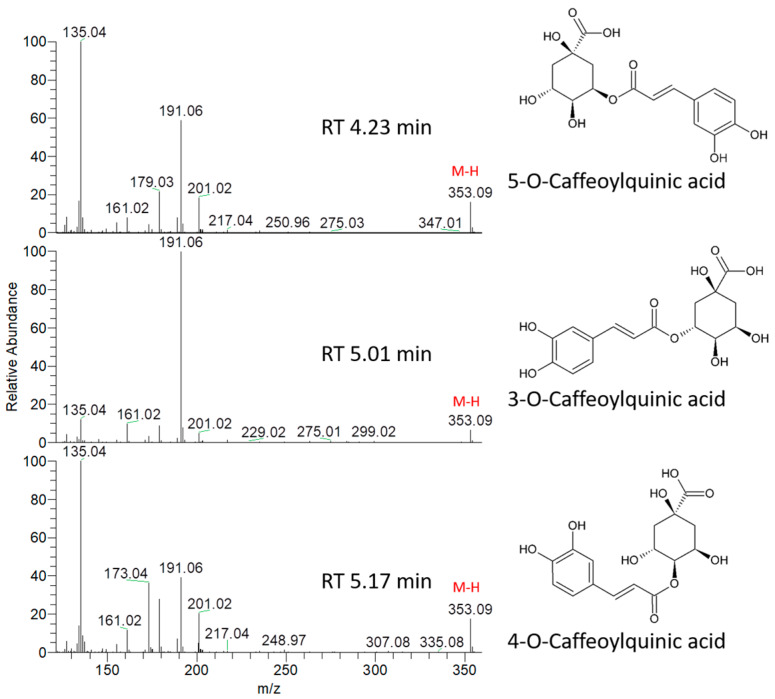
MS/MS spectrum of the [M − H]^−^ ions for the peaks at 4.23, 5.01 and 5.17 min representing caffeoylquinic acid isomers.

For 3-CQA (chlorogenic acid), the MS/MS spectrum showed a characteristic fragment of deprotonated quinic acid at *m*/*z* 191.05557 as a base peak, consistent with the published data of standard 3-CQA [18]. Consistently, very low abundance was detected for the fragments derived from deprotonated caffeic acid, caffeoyl decarbonylation, caffeoyl dehydration, caffeoyl dehydration and decarbonylation as well as quinic acid dehydration at *m*/*z* 179.03427, 135.04417, 161.02359, 133.02846 and 173.04462, respectively. Therefore, the peak at RT 5.01 min can be tentatively assigned to 3-CQA (Figure 2). The identity was also confirmed from online METLIN database (http://metlin.scripps.edu; Figure S1).

Additionally, for 4-CQA (cryptochlorogenic acid), stronger peaks for the quinic acid group at *m*/*z* 191.05557 and 173.04462 as well as the caffeoyl group at *m*/*z* 179.03427 and 135.04417 were detected (Figure 2). Therefore, the peak at RT 5.17 min can be tentatively assigned to 4-CQA (Figure 2). Meanwhile, for 5-CQA (neochlorogenic acid), stronger peaks were detected at *m*/*z* 191.05557, 179.03427 and 135.04417, however, the peak for the quinic acid group at *m*/*z* 173.04462 was very weak. Therefore, the peak at RT 4.23 min can be tentatively assigned to 5-CQA (Figure 2). Moreover, the chromatographic elution order for CQA was denoted as 5-CQA< 3-CQA < 4-CQA, consistent with the literature [18].

### 2.3. Effect of Extraction Solvent on Metabolite Abundance

**Figure 3:**
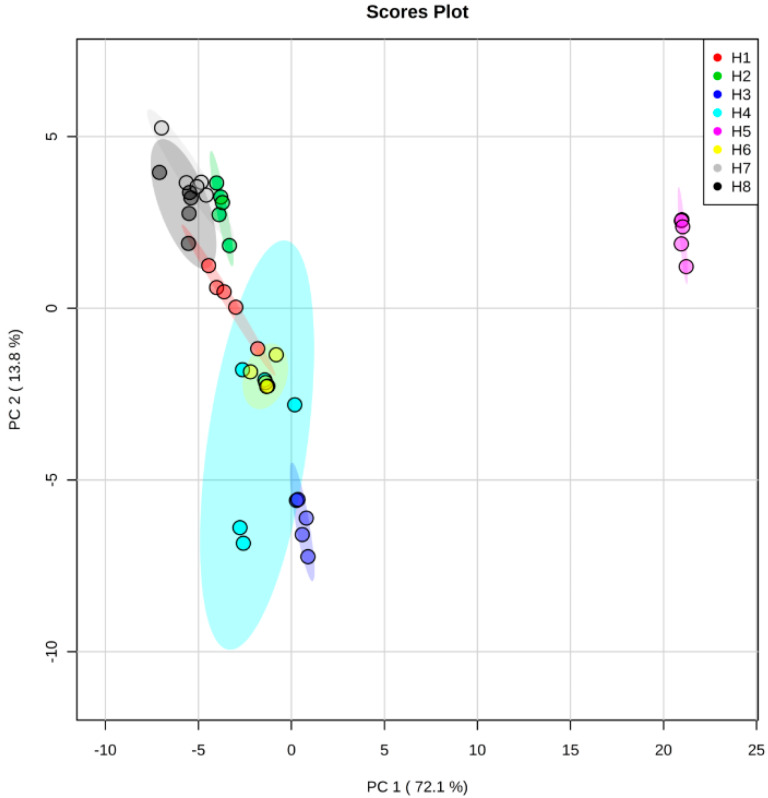
Principal component analysis (PCA) score plot of metabolites identified from *Hibiscus* calyces using different extraction methods. H1: 1% HCl in methanol, H2: 80% methanol, H3: 100% water, H4: 1% HCl in water, H5: 70% ethanol, H6: 50% methanol, H7: methanol and H8: 1% HCl in 70% ethanol.

**Figure 4:**
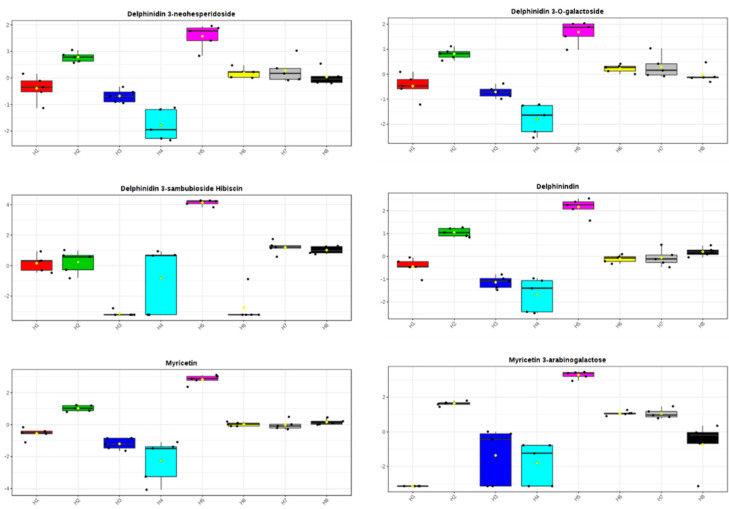
Metabolites with high abundance in *Hibiscus* calyces extracted with 70% ethanol (H5). The y-axis represents the log2 values of metabolite abundance.

To visualize the effect of the solvent on the metabolite changes, we performed a principal component analysis (PCA) of all extraction methods. PCA showed two distinct clusters representing different extraction methods (Figure 3). The first cluster contained *Hibiscus* calyces that were extracted with 70% ethanol (H5), which were separated from the second cluster of other extraction methods (Figure 3). The separation between these two clusters was along the PC1, which explained approximately 72% of the total variance. Furthermore, the PCA loading plot showed that the high level of delphinindin and its glycosides (delphinidin 3-neohesperidoside, delphinidin 3-*O*-galactoside, delphinidin 3-sambubioside (hibiscin) and myricetin and its glycoside (myricetin 3-arabinogalactose) contributed to the separation of samples extracted by 70% ethanol (H5) from other methods (Figure 4 and Figure S2). Additionally, organic acids (caffeoylshikimic acid, coumaroylquinic acid, caffeoylquinic acid, ethylchlorogenate, methyl chlorogenate and trimethylhydroxycitric acid), *Hibiscus* acid derivatives (*Hibiscus* acid dimethylester, *Hibiscus* acid hydroxyethyldimethylester and *Hibiscus* acid hydroxyethylester), flavonoids (kaempferol 3-*O*-glucuronide, Kaempferol-3-*O*-glucuronic acid methyl ester and kaempferol hexoside) and cleomiscosin have been detected as less predominant metabolites with lower concentration in samples extracted by 70% ethanol (H5; Figure S3).

### 2.4. Metabolites with the Highest ACE Inhibition Assay

Our previous results (Table 1) revealed that *Hibiscus* calyces extracted with 80% methanol (H2) showed the highest ACE inhibition capacity. Therefore, we investigated the 80% methanol extract for the abundant metabolites identified from the UPLC/MS analysis. As mentioned previously, the 70% ethanolic extract was clustered from other methods, although it did not show the highest ACE inhibition capacity indicating that its metabolites were not exclusively responsible for activity (Figure 4 and Figure S3).

**Figure 5:**
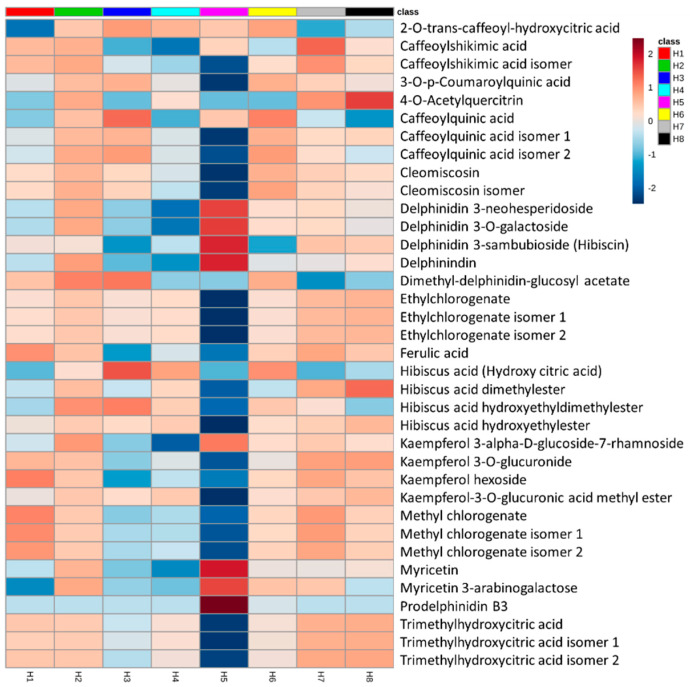
Heat map for the distribution of metabolites identified from *Hibiscus* extracts. The average of metabolite abundance from five biological replicates was used for the generation of heat maps. H1: 1% HCl in methanol, H2: 80% methanol, H3: 100% water, H4: 1% HCl in water, H5: 70% ethanol, H6: 50% methanol, H7: methanol and H8: 1% HCl in 70% ethanol.

We generated heat maps for the distribution of metabolites identified from different *Hibiscus* extracts (Figure 5). Interestingly, only samples extracted with 80% methanol (H2), with the highest ACE inhibition capacity, was predominated by almost all identified metabolites from all classes including flavonoids, anthocyanins, phenolic and organic acids (Figure 5). Additionally, no single metabolite was exclusively abundant in the 80% methanol (H2) extract. Moreover, no metabolites were missing compared to other methods. These results might indicate that the ACE inhibitory activity could not be represented by a single metabolite, rather the synergism of a group of detected metabolites. The group of metabolites did not have to be exclusively phenolic compounds since the 80% methanol (H2) did not show the highest percentage of total phenolics (Figure S4). The pure methanol extract (H7) had the highest phenolic content (95.80 ± 0.001 µg/mg), followed by H2, the 80% methanol extract that contained 86.83 ± 0.001 and H5, the 70% ethanol that contained 79.95 ± 0.001 µg/mg.

**Figure 6:**
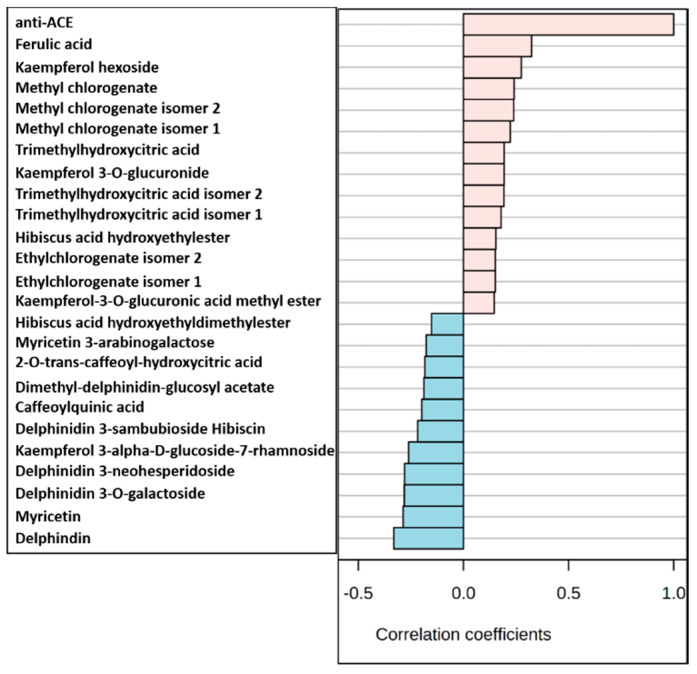
Top 25 metabolites correlated with angiotensin converting enzyme (ACE) inhibiting activity. Pearson’s correlation coefficients indicating the relationship between metabolites content and the ACE inhibition.

Intriguingly, the top metabolites that were positively correlated with ACE inhibiting activity included ferulic acid, kaempferol hexoside, methyl chlorogenate, trimethylhydroxycitric acid, kaempferol 3-*O*-glucuronide, trimethylhydroxycitric acid, *Hibiscus* acid hydroxyethylester and ethyl chlorogenate (Figure 6). Previous reports showed that carboxyl, hydroxyl and acrylic acid groups determine the ACE inhibitory activity of natural products [20]. The acrylic acid group existed in the compounds that were positively correlated with ACE inhibition (Figure 6). Among these compounds, ferulic acid, kaempferol glycosides, chlorogenate esters and citrate derivatives showed good positive correlation. These metabolites were also detected at higher levels in the 80% methanol extract (H2) confirming the highest ACEI and the distinctive clustering of H2 (Figure 5, Figures S5 and S6). Since the 80% methanol extract was found to have the highest ACE inhibitory activity, we evaluated the availability of preparing enriched fraction or isolated compound (s) with the highest ACEI potential.

## 3. Discussion

Cardiovascular diseases (CVDs) constitute a serious problem with nearly a 30% estimated mortality rate worldwide. Despite the advancements in diagnosis and treatment of cardiovascular diseases, their incidence is still rising. The most frequent CVDs include hypertension, atherosclerosis, angina pectoris, ischemic heart disease and congestive heart failure. Hypertension is a serious risk factor for the progression of angina pectoris, and congestive heart failure. More than 950 million people worldwide are estimated to be hypertensive [21]. Hypertension can be classified as primary/essential or secondary. The major causes of primary hypertension include family history, high salt consumption and obesity, on the other hand, kidney and adrenal diseases accounts for secondary hypertension [22].

Antihypertensive drugs can be classified into two major groups, the first group includes drugs that can directly or indirectly block the renin–angiotensin system (RAS) and thus cause vasodilatation such as direct renin inhibitors (DRIs), angiotensin receptor antagonists (ARAs), β-blockers and angiotensin-converting enzyme inhibitors (ACEIs). The second group includes drugs that increase water/sodium excretion such as diuretics and calcium channel blockers (CCBs) [23]. ACEIs are considered a first-line treatment in primary hypertension owing to their cardiac protective effects, so they are also recommended in patients suffering from post-myocardial infarction, heart failure and diabetic nephropathy [24,25]. Interestingly, ACEIs have been historically discovered from a natural source, the venom of the Brazilian pit viper, and this discovery led to the development of, orally-active synthetic ACEIs [25].

Despite the advancements in treatment of hypertension, the incidence rate of the disease is progressively still rising. Therefore, new strategies of medications are still needed. Natural products constitute an indispensable source for prevention and treatment of hypertension. Among plants, *Nigella sativa, Beta vulgaris, Coptis chinensis, Crataegus monogyna, Allium sativum, Crocus sativus, Hibiscus sabdariffa* and *Cymbopogon citratus* have been reported as antihypertensive herbs [26]. *Hibiscus sabdariffa* L. extract has been reported to be safe in ordinary doses with no evidence of toxicity [4].

The antihypertensive activity of *Hibiscus sabdariffa* L. has been excessively reported. *Hibiscus* antihypertensive effects have been related to its anthocyanins and hibiscus acid [27]. In vivo clinical trials showed that a daily consumption of *H. sabdariffa* tea decreased the blood pressure compared to the control placebo, with no significant adverse clinical effects [28]. The proposed mechanism of *Hibiscus* antihypertensive activity could be attributed to its vasorelaxant effect through increasing nitric oxide production, Ca^2+^ channel blocking activity as well as angiotensin converting enzyme (ACE) inhibitor activity [8,10,16]. Additionally, *Hibiscus* extract has been shown, next to its blood pressure lowering effect, to reduce the heart rate via a negative chronotropic action salt-sensitive hypertension and hypertension due to chronic NOS inhibition in animal models [29].

The main goal of this study was to provide a comprehensive profiling of *Hibiscus* calyces in response to different extraction solvents. The composition of each extract obtained from a different extraction solvent was assessed via UPLC-HRMS. This comprehensive analysis allowed the reliable identification of several compounds covering *Hibiscus* secondary metabolite classes such as aliphatic organic acids (i.e., *Hibiscus* acid), phenolic acids (caffeoylquinic acids), flavonoids and anthocyanins.

Previous phytochemical studies on *Hibiscus* have shown that the calyces are rich in organic acids (*Hibiscus* acid and hydroxycitric acid), phenolic acids (caffeoylquinic acid), fatty acids (linoleic acid), flavonoids (gossypetin, myricetin, hibiscetin, kaempferol and quercetin) and anthocyanins (hibiscin) [13,14]. Phenolic acid such as ferulic and chlorogenic acids, organic acids such as citrate derivatives and flavonoids such as kaempferol have been reported to have antihypertensive potential [18,20,30]. Therefore, we argued from our results that the ferulic and chlorogenic acids as well as citrate derivatives and the flavonoid kaempferol might significantly contribute synergistically to the ACE inhibitory activity of the 80% methanol extract (H2). The presence hydroxyl, carboxyl and acrylic acid functional groups in the structure of phenolic acids such as chlorogenic acid is a key determinant of their ability to inhibit ACE. The higher number of hydroxyl groups in chlorogenic acid make it more active than caffeic acid [31].

Interestingly, from our eight extraction solvents that have been used to screen for the highest content of metabolites that inhibit ACE, the 80% methanolic extract showed the highest ACE inhibition, followed by the 100% water. Therefore, we expected other extraction solvents containing different proportions of methanol/water such the 50% methanol or also the 100% methanol might have similar extraction efficiency for ACE-inhibitory metabolites. However, the 100% methanol was far from the 80% methanol for its ACE-inhibitory activity. Therefore, we hypothesized that the presence of certain proportion of water might be essential for the efficient extraction of ACE-inhibitory metabolites. On the other side, it seemed that the percentage of water should be optimal for the efficient extraction since the 50% methanol, which only contained a high proportion of water, showed the least activity among all extraction methods. This contradicts the ability of the 100% water to efficiently extract ACE-inhibitory metabolites. Although, we did not have a clear explanation for this contradiction, we could speculate from the metabolites data. When we performed the PCA analysis including the only extraction methods containing methanol, water or both (viz. 100% methanol, 80% methanol, 50% methanol and 100% water), we could detect a nice pattern for the clustering of samples (Figure S7). From the PCA analysis, samples representing the 100% methanol was clustered from the 80% methanol followed by the 50% methanol and farthest from the 100% water. This linear pattern indicates that the different proportions of methanol/water varied quantitative extraction efficiency, considering that we could not detect qualitative differences between the four methods in terms of the number of metabolites and only the variations were quantitative. Looking at the relative abundance of the metabolites involved in clustering of samples (Figures S8 and S9), we have detected a group of metabolites including kaempferol hexoside, kaempferol 3-*O*-glucuronide and ferulic acid showed the highest abundance in the presence of high methanol proportion. Other metabolites such as delphindin, myricetin as well as their glycosides showed the optimum abundance in 80% methanol. In addition, metabolites such as *Hibiscus* acid derivatives and kaempferol derivatives showed the least abundance in 50% methanol. These different patterns of metabolites among the different extraction solvents indicate that the optimum solvent for extraction should preferentially allow the highest recovery of metabolites that synergistically act to inhibit ACE. For instance, the 50% methanol showed a significantly higher level of total phenolics than the 100% water (Figure S4), also, the highest abundance of the phenolic coumarino-lignans, cleomiscosin and its isomer (Figure S10). This result indicates that the total phenolics in general could not be coincided a marker for the ACE-inhibitory activity and the presence of the optimum amount of specific compounds is the driving factor. The presence of compounds that may activate ACE and decrease the ability of others that target the enzyme could not be excluded. Future experiments such as tracing the metabolites that can effectively inhibit ACE after fractionation of the total extracts will be indispensable for benchmarking the metabolites with ACE-inhibitory activity.

In conclusion, a comprehensive study was undertaken to investigate the suitable extraction solvent for the possible in vitro benefits of *Hibiscus* calyces in hypertension. The 80% methanol and 100% water showed the highest in vitro ACE inhibitory activity, among other selected extraction solvents. Intriguingly, *Hibiscus* herbal teas are prepared in hot water, confirming the ethnopharmacological knowledge of *Hibiscus* having the potential for the management of hypertension. The promising results of the activity and the metabolite content of 80% methanol deserve future work. First to perform biologically guided fractionation of this extract to find the exact fraction that have the ACEI activity. If the obtained results are encouraging, further studies about the isolation and identification of the active principles, and to evaluate the possible synergism among fraction components. Second, the antihypertensive effect of *Hibiscus* calyces extract/fraction in vivo assays has to be performed, to find the possible underlying mechanism. Our main intention here was to encourage innovative research based on prior ethnopharmacological knowledge about *Hibiscus* antihypertensive activity. We do not, of course, encourage the community to use 80% methanol for consumption. Alternatively, the herbal teas should be prepared in aqueous solutions.

## 4. Materials and Methods

### 4.1. Chemicals

All chemical reagents and extraction solvents were of analytical grade and all analysis solvents were of HPLC grade. The angiotensin converting enzyme (from rabbit lung), histidine-l-hippuryl-l-leucine-chloride (HHL), Folin–Ciocalteu reagent, gallic acid and methanol were purchased from Sigma-Aldrich, Darmstadt, Germany. Borate saline buffer components (Boric acid, KCL and NaOH), dimethyl sulfoxide (DMSO), tris HCl and NaCl were purchased from Merck, Darmstadt, Germany.

### 4.2. Plant Material and Extraction

The calyces of *Hibiscus sabdariffa* L. (HS) were obtained from HCA products Sdn Bhd. during Spring 2015. The plant was kindly identified in the Forest Research institute, Petaling Jaya, Malaysia. A voucher specimen (7-06-2015) was kept in the herbarium of Pharmacognosy Department, Faculty of Pharmacy, Cairo University, Cairo, Egypt. The air-dried powdered calyces of HS (50 g) were extracted using H1: 1% HCl in methanol, H2: 80% methanol, H3: 100% water, H4: 1% HCl in water, H5: 70% ethanol, H6: 50% methanol, H7: methanol and H8: 1% HCl in 70% ethanol. The extraction was done in a three-stage procedure (each in a solvent-sample ratio of 20:1), each time continued for 30 min using ultrasonic bath at 60 °C. It was reported that the use of 1% HCl aids the extraction of anthocyanins through the plant cell membranes denaturation, preventing their oxidation as anthocyanins are more stable and in acidic pH [32]. The combined extracts for each solvent were evaporated under reduced pressure using rotary evaporator (at 40 °C) to yield solid residues weighing H1: 35.69 g, H2: 24.35 g, H3: 27.96 g, H4: 32.61 g, H5: 22.53 g, H6: 25.10 g, H7: 22.23 g and H8: 37.27 g.

### 4.3. Quantitative Estimation of the Total Phenolic Content

Spectrophotometric determination of the total phenolic content of the extracts was carried out using the Folin–Ciocalteu colorimetric method [33]. A stock solution of gallic acid was prepared at a concentration 1 mg/mL and a standard calibration curve was established using serial dilutions ranging from 20 to 280 μg/mL in methanol. To 1 mL of each dilution of gallic acid, 10 mL of distilled water, 1.5 mL of Folin–Ciocalteu reagent and 4 mL of 20% sodium carbonate solution were added and the volume was completed to 25 mL with distilled water. The extracts were dissolved in methanol at a concentration of 1 mg/mL and 1 mL of each extract was tested as above. All samples were incubated for 30 min. at room temperature protected from light. The absorbance was measured at 760 nm using a spectrophotometer (Shimadzu UV-1650PC, Shimadzu EuropaGmbH, Duisberg, Germany) against a blank (methanol instead of the test solution). Three replicates were carried out for each concentration.

### 4.4. ACE Inhibition Assay

The experimental model used for the assay was the inhibition of the angiotensin conversion enzyme (ACE), using a chromogenic synthetic substrate, histidine-l-hippuryl-l-leucine-chloride (HHL). The method depends on His–Leu formation by the cleavage of HHL in the presence of ACE. The formation of His–Leu was measured using the fluorescence method. The ACE inhibition potential of the different plant extracts was tested according to [34] with minor modifications. The different extracts were dissolved in methanol: water (1:1) to prepare stock solution of each extract (100 mg/mL). Serial dilutions were prepared for each test solution at concentrations 0.1, 1, 10 and 100 mg/mL.

The test solutions were prepared by adding 40 µL enzyme solution (2 mU ACE prepared in 0.1 M Na borate buffer) to 20 µL of each tested dilution of each sample and then incubated at 37 °C for 10 min. then, 40 µL HHL substrate (0.8 mM/L) were added to the solution. The test solutions were all incubated at 37 °C for 1 h and 60 µL 0.5 M sodium hydroxide were then added to stop the reaction. Blank solutions were prepared for each sample by adding buffer solution instead of the enzyme solution. The control solution was prepared by using each specified solvent instead of the sample. Triplicates were run for each sample. Experiments were set in 96 well microplates. The formation of His–Leu was measured using the florescence method, using the FLUOstar OPTIMA plate reader (BMG Labtech Inc., Offenburg, Germany) at excitation and emission wavelengths of 360 nm and 500 nm, respectively. The percentages of inhibition (% I ± SD) were calculated using the following formula:

% Inhibition = [Fl control − (Fl sample − Fl blank)/Flcontrol] × 100(1)

The IC50 ± SD (*n* = 3) values were calculated in mg/mL by linear interpolation.

### 4.5. UPLC-MS/MS Analysis

The metabolites of extracts were separated on RP High Strength Silica (HSS) T3 C18 column (100 mm × 2.1 mm containing 1.7 μm diameter particles, Waters), using a Waters Acquity UPLC system. The injection volume was two µL and the flow rate was adjusted to 400 µL/min. The mobile phases used for chromatographic separation were water containing 0.1% formic acid (Buffer A) and acetonitrile containing 0.1% formic acid (Buffer B). The following gradient was applied: 1 min 1% B, 13 min linear gradient from 1% B to 35% B, 14.5 min linear gradient from 35% B to 70% B, 15.5 min linear gradient from 70% B to 99% B, hold 99% B until 17 min, 17.5 min linear gradient from 99% B to 1% B and re-equilibrate the column for 2.5 min. The mass spectra were acquired by full scan MS in negative ionization mode on an exactive high resolution Orbitrap-type MS (Thermo Fisher, Bremen, Germany) [35]. The spectra were recorded, covering a mass range 100–1500 *m*/*z*, using alternating full-scan and all-ion fragmentation-scan modes. The resolution was set to 10,000, with 10 scans per second. The capillary voltage was set to 3 kV with a sheath gas flow value of 60 and an auxiliary gas flow of 35. The capillary temperature was set to 150 °C, whereas the drying gas in the heated electrospray source was set to 350 °C. Chromatograms alignment was performed by using the Progenesis QI software package (Progenesis QI Version 2.2, Nonlinear Dynamics, Newcastle, UK). All the obtained data were analyzed and correlated using metaboanalyst (https://www.metaboanalyst.ca/).

## 5. Conclusions

The choice of a proper extraction solvent is necessary to obtain the desired concentration of the secondary metabolites and subsequently the desired biological activity. In our study, 80% methanol extract of *H. sabdarifa* calyces had the powerful ACEI activity. Analyzing the metabolome of the different extracts using UPLC-MS/MS and PCA analysis showed that the detected compounds in 80% methanol played a significant role in its activity especially ferulic acid, kaempferol glycosides, chlorogenate esters and citrate derivatives. Our future prospective is to perform the in vivo testing for the eight extract in a hypertensive model in rats.

## Figures and Tables

**Table 1:** Results of the angiotensin converting enzyme inhibition assay.

The Tested Sample	IC50 (µg/mL)
H1	6.293 ± 0.03896
H2	0.01255 ± 0.00343
H3	0.2058 ± 0.05045
H4	9.217 ± 1.0150
H5	0.6390 ± 0.032
H6	>200
H7	6.058 ± 0.084
H8	8.025 ± 1.501
Captopril standard drug	0.210 ± 0.005

H1: 1% HCl in methanol, H2: 80% methanol, H3: 100% water and H4: 1% HCl in water, H5: 70% ethanol, H6: 50% methanol, H7: methanol and H8: 1% HCl in 70% ethanol.

**Table 2:** Peak assignments of metabolites using ultra-performance liquid chromatography (UPLC)-MS/MS in the negative mode.

Peak No.	RT	Formula	[M − H]^−^	rdb	Error	Mass Fragmentation	The Identified Compounds
1	0.74	C_6_H_7_O_8_	207.01407	3.5	2.543	189, 127	Hydroxy citric acid
2	1.48	C_10_H_16_O_8_	263.04074	4.5	3.73	221.03,203.02,189, 185.01,127	*Hibiscus* acid hydroxyethyldimethylesther
3	2.64	C_8_H_11_O_8_	235.04575	3.5	3.856	189.0, 169.01, 127	*Hibiscus* acid hydroxyethylesther
4	3.12	C_18_H_17_O_14_	475.07144	10.5	−1.469	405.03,363.06,285.02, 235.05,217.03,199.02,189,152.98,111.04	Kaempferol-3-*O*-glucuronic acid methyl esther
5	3.91	C_9_H_14_O_8_	249.0614	6.5	−3.069	307.02, 249.06,206.97, 203.02, 185.01, 127	Trimethylhydroxycitric acid
6	4.23	C_16_H_17_O_9_	353.08823	8.5	4.309	347.06, 305.07, 191.06, 112.98	Caffeoylquinic acid
7	4.46	C_9_H_14_O_8_	249.06	6.5	−3.069	307.02, 249.06,206.97, 203.02, 185.01, 127	Trimethylhydroxycitric acid isomer
8	4.60	C_26_H_27_O_16_	595.1319	11.5	−1.1	521.11,485.51,419.05,334.04,300.03,249.06,217.03,189,	Delphinidin 3-sambubioside (Hibiscin)
9	4.71	C_9_H_14_O_8_	249.0614	6.5	−3.069	307.02, 249.06,206.97, 203.02, 185.01, 127	Trimethylhydroxycitric acid isomer
10	5.01	C_16_H_17_O_9_	353.08826	8.5	4.394	263.08, 217.03, 191.06, 145.05	Caffeoylquinic acid isomer
11	5.17	C_16_H_17_O_9_	353.08823	8.5	4.039	191.06, 135.04	Caffeoylquinic acid isomer
12	5.18	C_15_H_14_O_11_	369.0464	4.6	1.10	189, 135,127	2-*O*-trans-Caffeoyl-hydroxycitric acid
13	5.42	C_21_H_19_O_11_	447.05923	13.5	7.667	369.08, 299.02, 189, 179.03, 135.04, 112.98	Kaempferol hexoside
14	5.60	C_17_H_19_O_9_	367.10385	8.5	4.063	191.06, 161.02, 133.03	Methyl chlorogenate
15	5.66	C_8_H_9_O_7_	217.03494	4.5	3.045	199.02, 189, 152.98, 111.04	*Hibiscus* acid dimethylesther
16	5.84	C_16_H_17_O_8_	337.09329	8.5	4.438	304.91, 242.94, 214.93, 193.05, 189, 173.04, 163.04, 112.98	3-*O*-p-Coumaroylquinic acid
17	5.94	C_26_H_27_O_17_	611.1259	6.5	−3.069	317, 315	Myricetin 3-arabinogalactoside
18	6.28	C_17_H_19_O_9_	367.10382	8.5	3.981	362.88, 174.96, 161.02, 133.03, 112.98	Methyl chlorogenate (isomer)
19	6.36	C_21_H_18_O_12_	461.0750	6.6	2.19	285.1, 180	Kaempferol 3-*O*-glucuronide
20	6.39	C_16_H_15_O_8_	335.07773	9.5	4.734	248.96, 214.93, 174.96, 161.02, 133.03	Caffeoylshikimic acid
21	6.53	C_16_H_15_O_8_	335.07764	9.5	4.465	304.91, 214.93, 174.96, 161.02, 112.98	Caffeoylshikimic acid isomer
22	6.61	C_17_H_19_O_9_	367.10388	8.5	4.144	135, 179	Methyl chlorogenate (isomer)
23	6.63	C_18_H_21_O_9_	381.11957	8.5	4.097	360.06, 316.95, 206.97, 174.96	Ethylchlorogenate
24	6.85	C_27_H_30_O_16_	609.14734	5.6	4.144	486.14, 367.10,300.03, 248.96, 214.93, 189, 174.96	Delphinidin 3-neohesperidoside
25	7.08	C_21_H_19_O_12_	463.08905	12.5	4.206	304.91, 300.03, 271.03, 207.07, 189.0, 174.96, 129.97	Delphinidin 3-*O*-galactoside
26	7.24	C_18_H_21_O_9_	381.11957	8.5	4.097	360.06, 316.95, 206.97, 174.96	Ethylchlorogenate isomer
27	7.41	C_27_H_29_O_15_	593.15186	13.5	2.973	486.07, 381.13, 316.95, 285.04, 214.93, 189.0, 74.96, 129.97, 112.98	Kaempferol 3-alpha-d-glucoside-7-rhamnoside
28	7.51	C_18_H_21_O_9_	381.11957	8.5	4.097	360.06, 316.95,206.97, 174.96	Ethylchlorogenate isomer
29	8.17	C_15_H_10_O_8_	317.0302	3.6	−2.90	248.9644, 189.0064	Myricetin
30	8.53	C_10_H_9_O_4_	193.05009	6.5	2.874	174.96,133.03	Ferulic acid
31	8.68	C_23_H_22_O_12_	489.1062	1.9	2.67	301.12	4′′-*O*-Acetylquercitrin
32	8.78	C_20_H_18_O_8_	385.0931	2.9	−1.1		Cleomiscosin
33	8.86	C_30_H_26_O_14_	609.1260	10.1	2.81	301.01	Prodelphinidin B3
34	9.30	C_20_H_18_O_8_	385.0931	7.1	2.98		Cleomiscosin isomer
35	9.48	C_15_H_9_O_7_	301.03558	11.5	4.322	260.93, 235.93, 206.93, 174.96, 121.03, 116.93	Delphinidin
36	12.21	C_24_H_37_O_13_	533.22485	6.5	3.718	486.25,379.16,339.20,311.17,183.01	Dimethyl-delphinidin-glucosyl acetate

## Supplementary Materials

The following are available online. Figure S1: Identification of chlorogenic acid based on MS/MS spectrum in comparison to metlin database. The upper panel represents MS spectrum obtained from our analysis; the lower panel represents the data in metlin database online (http://metlin.scripps.edu). Figure S2. Principal component analysis (PCA) loading plot of metabolites identified from *Hibiscus* calyces using different extraction methods. Figure S3: Metabolites with low abundance in *Hibiscus* calyces extracted with 70% ethanol (H5). Log2 fold change of metabolite abundance has been used for the boxplots. Figure S4: Total phenolic contents of the extracts. Figure S5. Metabolites that were detected at higher level in 80% MeOH *Hibiscus* extracts. Figure S6: Dendrogram of the investigated extraction method based on the identified LC/MS metabolites.
