# Supplementary material for: Optimization of an Extraction Solvent for Angiotensin-Converting Enzyme Inhibitors from Hibiscus sabdariffa L. Based on Its UPLC-MS/MS Metabolic Profiling
Source: Molecules. 2020 May 14;25(10):2307. doi: 10.3390/molecules25102307 (PMC7287694; doi:10.3390/molecules25102307)
Supplement: Supplementary file 1 [file molecules-25-02307-s001.pdf]

Optimization of an Extraction Solvent for Angiotensin-Converting Enzyme Inhibitors from *Hibiscus sabdariffa* L. Based on its UPLC-MS/MS Metabolic Profiling

Mohamed A. Salem [1], Haidy E. Michel [2], Marwa I. Ezzat [3], Mona M. Okba [3], Ahmed M. EL-Desoky [4], Shanaz O. Mohamed [5] and Shahira M. Ezzat [3,6,*]

[1] Department of Pharmacognosy, Faculty of Pharmacy, Menoufia University, Gamal Abd El Nasr st., Shibin Elkom 32511, Egypt; mohamed.salem@phrm.menofia.edu.eg

[2] Department of Pharmacology and Toxicology, Faculty of Pharmacy, Ain-Shams University, Cairo 11566, Egypt; heidieffat@pharma.asu.edu.eg

[3] Pharmacognosy Department, Faculty of Pharmacy, Cairo University, Kasr El-Ainy Street, Cairo 11562, Egypt; marwa.ezzat@pharma.cu.edu.eg (M.I.E.); mona.morad@pharma.cu.edu.eg (M.M.O.)

[4] Department of Molecular Biology, Genetic Engineering and Biotechnology Research Institute (GEBRI), University of Sadat City (USC), Sadat City 32958, Egypt; ahmed.desoky@mynaturalwellness.com

[5] School of Pharmaceutical Sciences, Universiti Sains Malaysia, Penang 11800, Malaysia; shanaz.mohamed@mynaturalwellness.com

[6] Department of Pharmacognosy, Faculty of Pharmacy, October University for Modern Sciences and Arts (MSA), Giza 12451, Egypt

* Correspondence: shahira.ezzat@pharma.cu.edu.eg; Tel.: +20-120-000-4301

Received: 15 March 2020; Accepted: 16 April 2020; Published: 17 April 2020

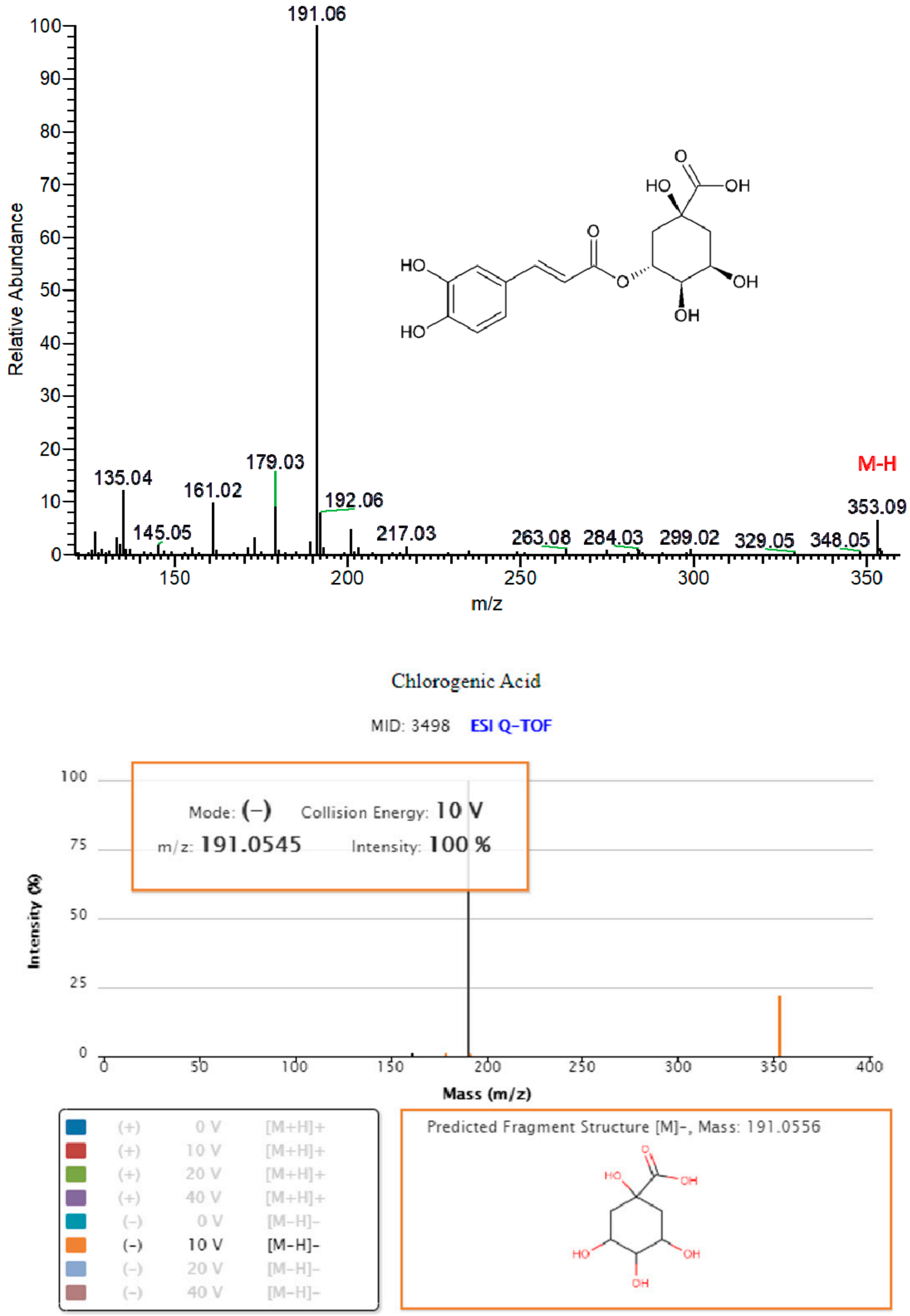

Figure S1. Identification of chlorogenic acid based on MS/MS spectrum in comparison to metlin database. The upper panel represents MS spectrum obtained from our analysis; the lower panel represents the data in metlin database online (http://metlin.scripps.edu).

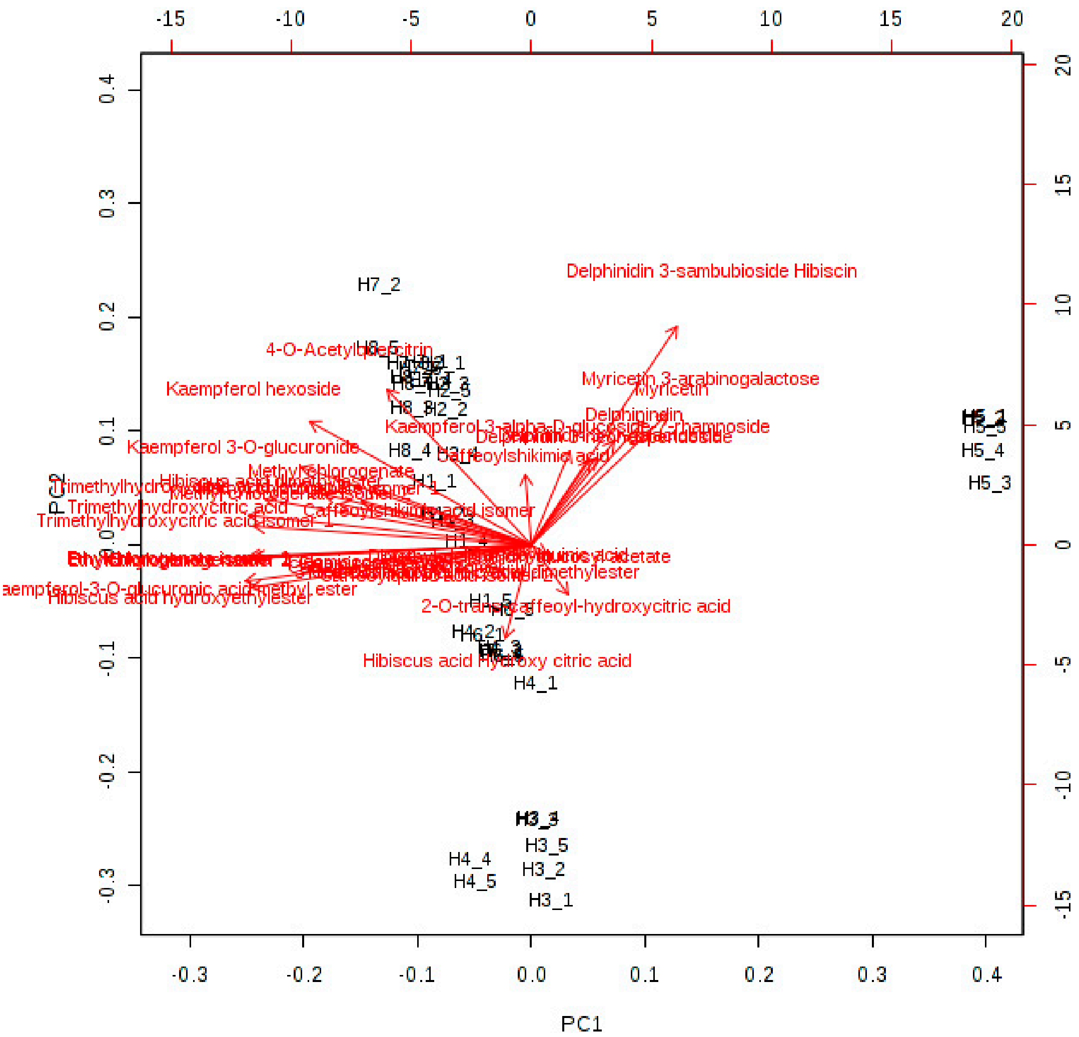

Figure S2. Principal component analysis (PCA) loading plot of metabolites identified from Hibiscus calyces using different extraction methods. H1: 1% HCl in methanol, **H2**: 80% methanol, **H3**: 100% water, **H4**: 1% HCl in water, **H5**: 70% ethanol, **H6**: 50% methanol, **H7**: methanol and **H8**: 1% HCl in 70% ethanol. Loadings: describe the relationships between variables and plotted with red lines**.** Variables with high loadings; this indicates that the loading is interpretable

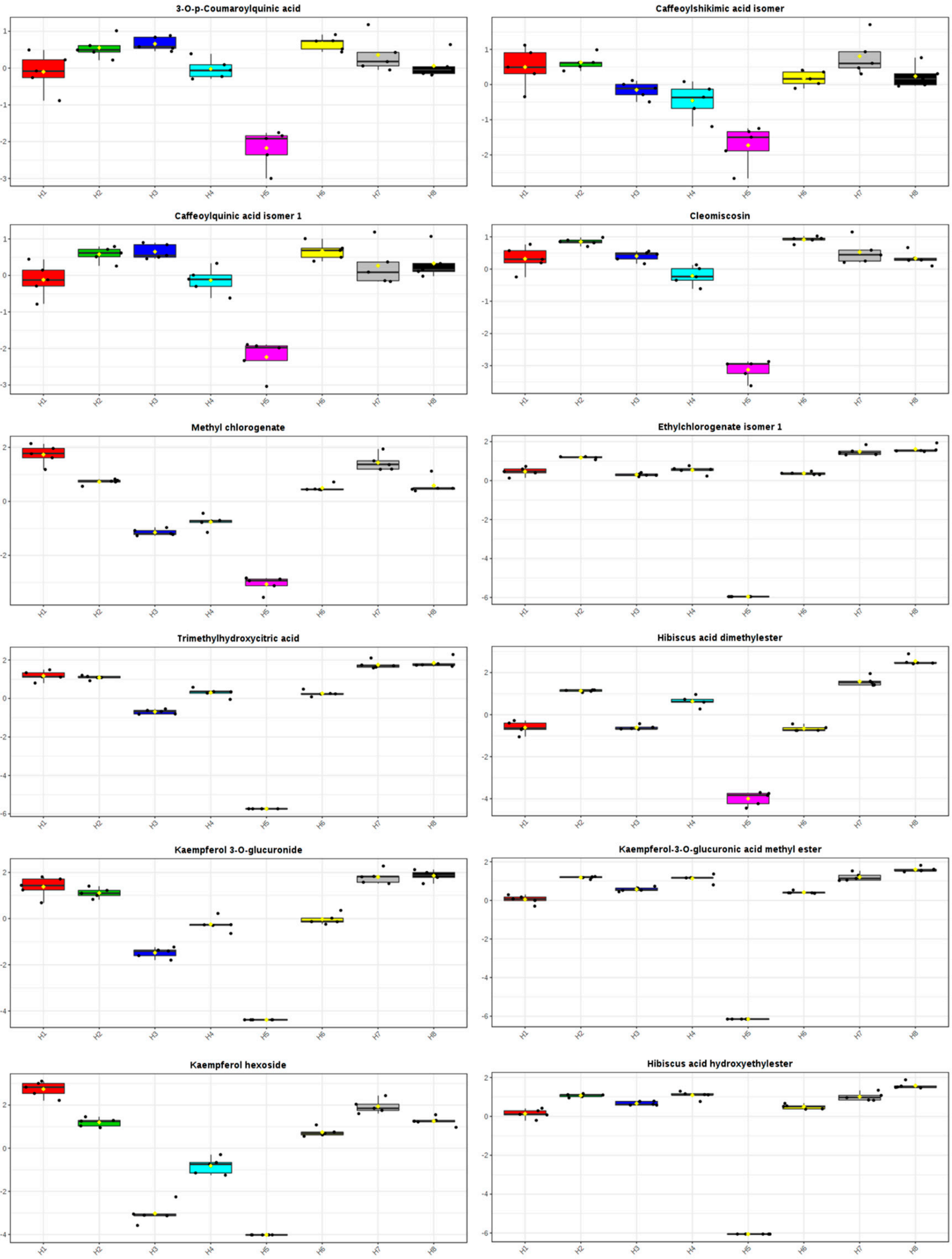

Figure S3. Metabolites with low abundance in Hibiscus calyces extracted with 70% ethanol (H5). The y-axis represents the $\log_2$ value of metabolite abundance

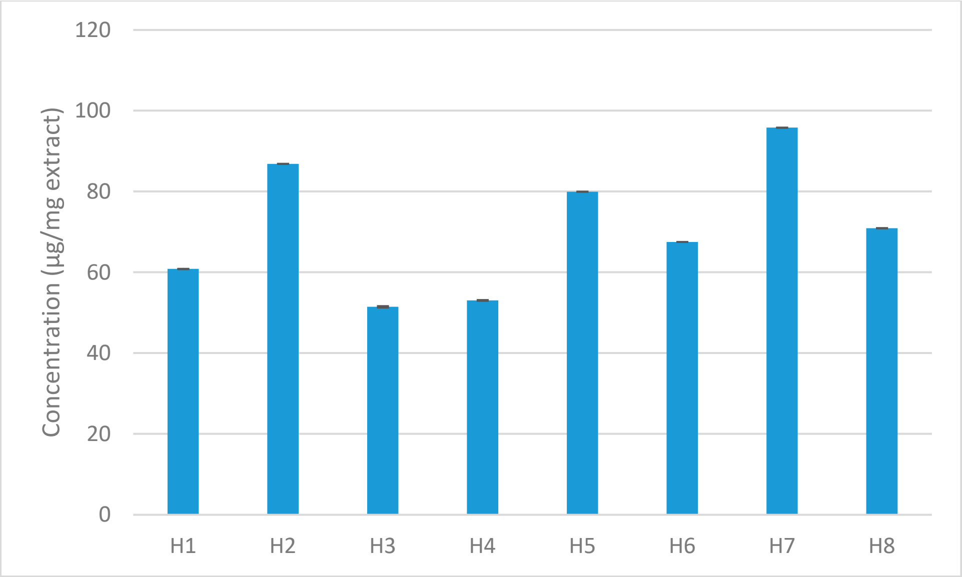

Figure S4. Total phenolic contents of the extracts

H1: 1% HCl in methanol, **H2**: 80% methanol, **H3**: 100% water and **H4**: 1% HCl in water, **H5**: 70% ethanol, **H6**: 50% methanol, **H7**: methanol and **H8**: 1% HCl in 70% ethanol

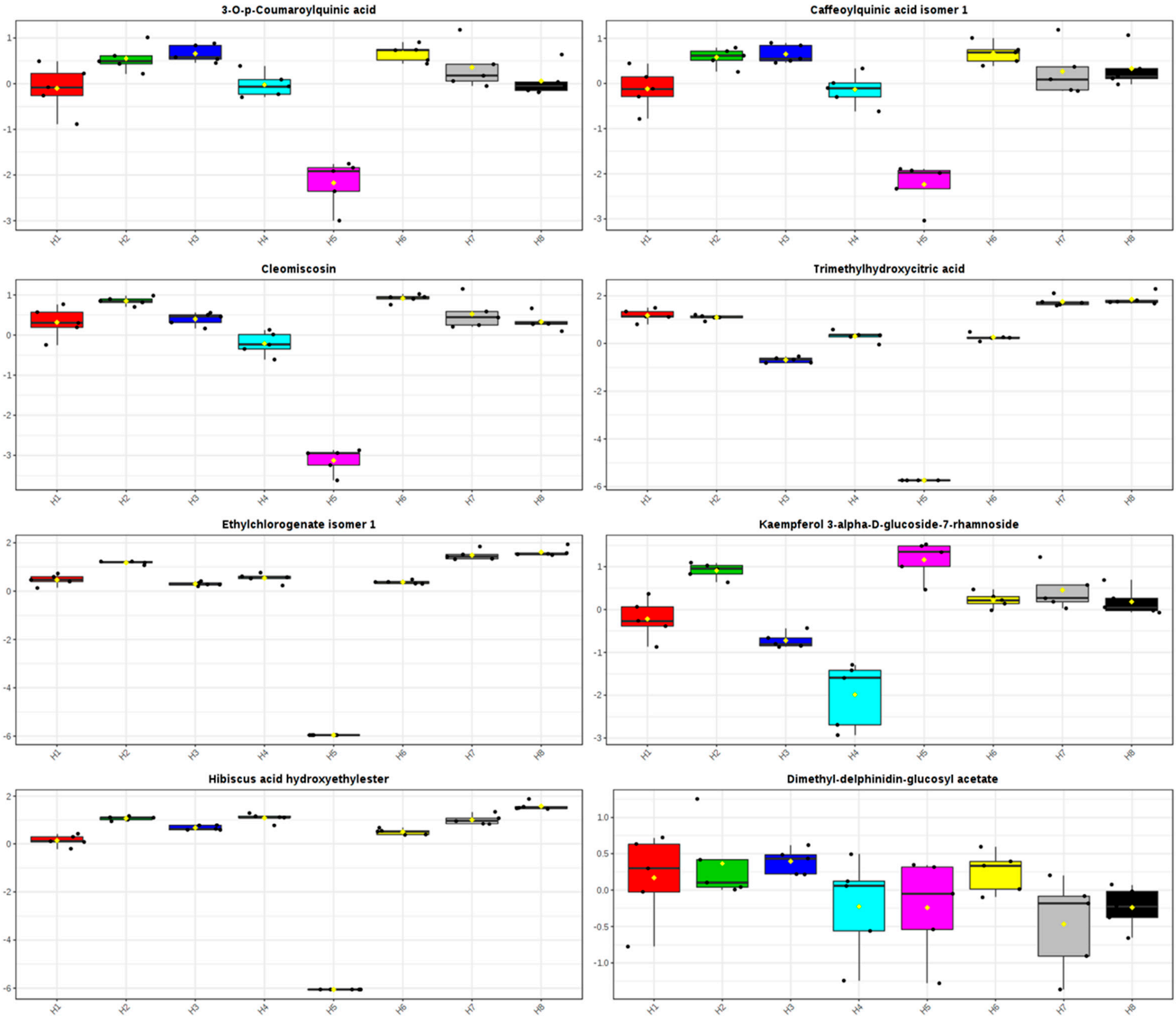

Figure S5. Metabolites that were detected at higher level in 80% MeOH Hibiscus extracts.
The y-axis represents the log_2 value of metabolite abundance

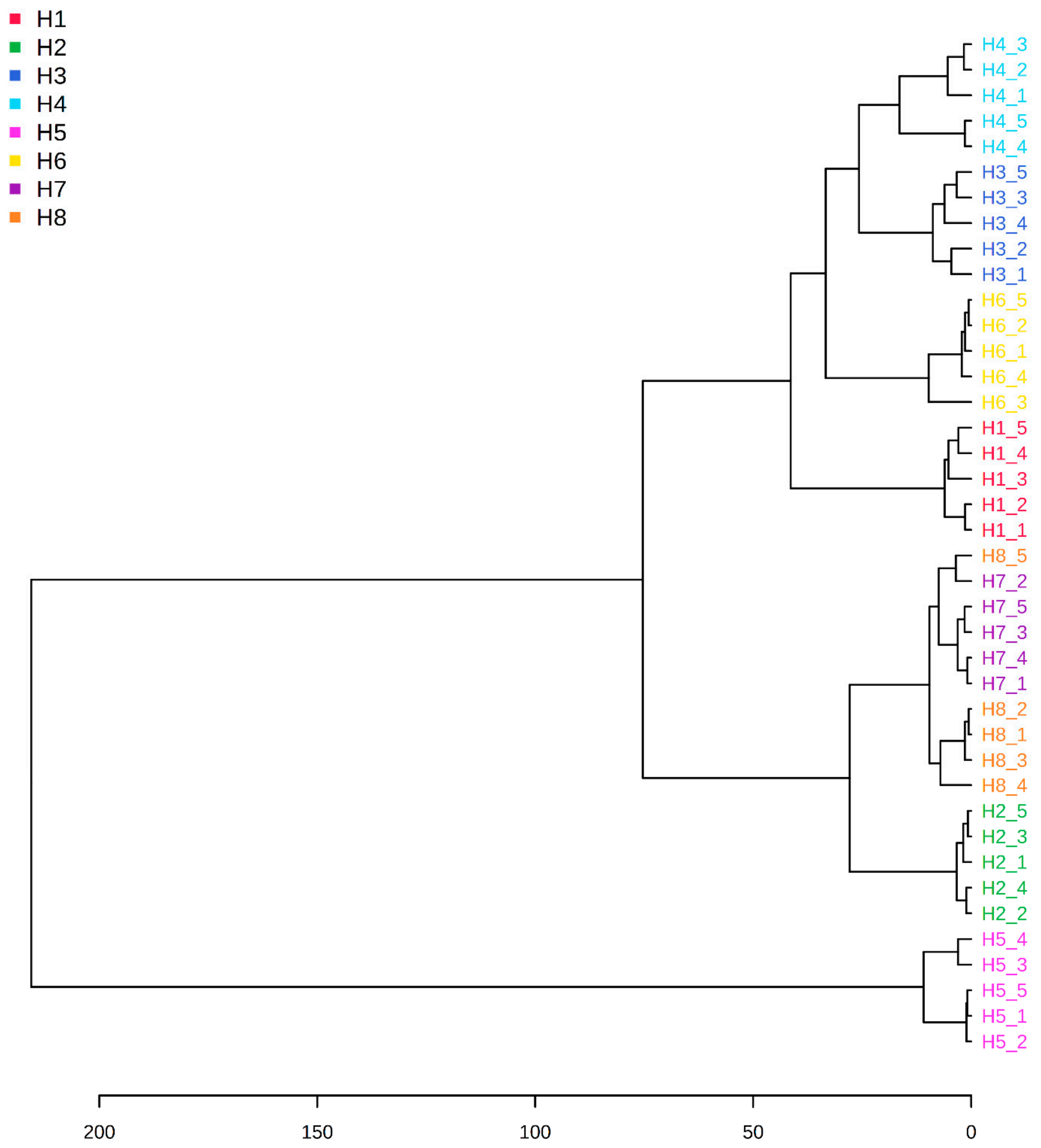

Figure S6: Dendrogram of the investigated extraction method based on the identified LC/MS metabolites. The dendrogram is a diagram that shows the hierarchical relationship between the different extraction methods. The horizontal axis of the dendrogram represents the distance or dissimilarity between clusters. The vertical axis represents the objects (extraction method) and clusters. In this case, the dendrogram shows us that the big difference was observed between H5 and other clusters.

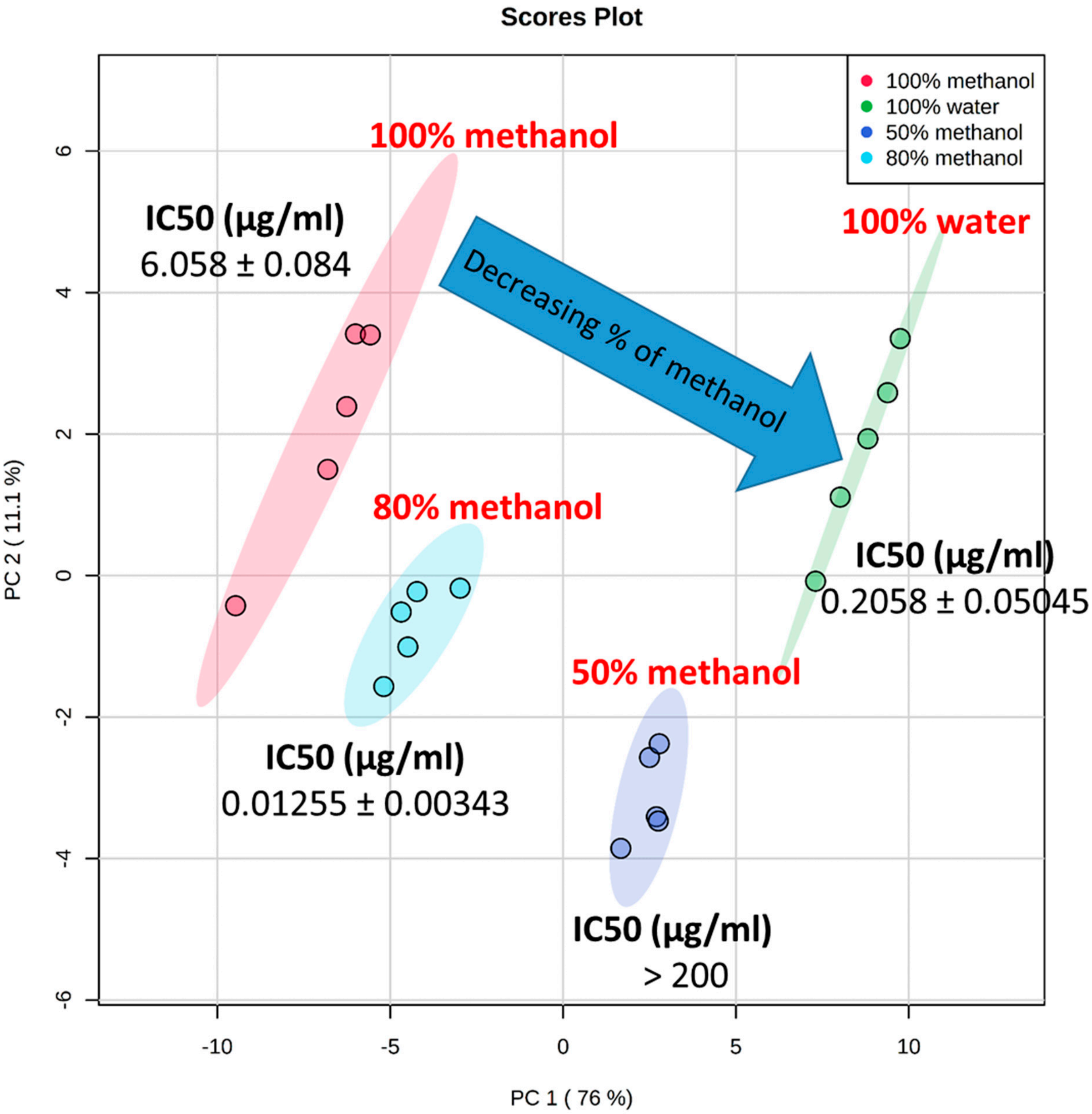

Figure S7. Principal component analysis (PCA) score plot of metabolites identified from Hibiscus calyces using different extraction methods with different proportions of methanol/water.

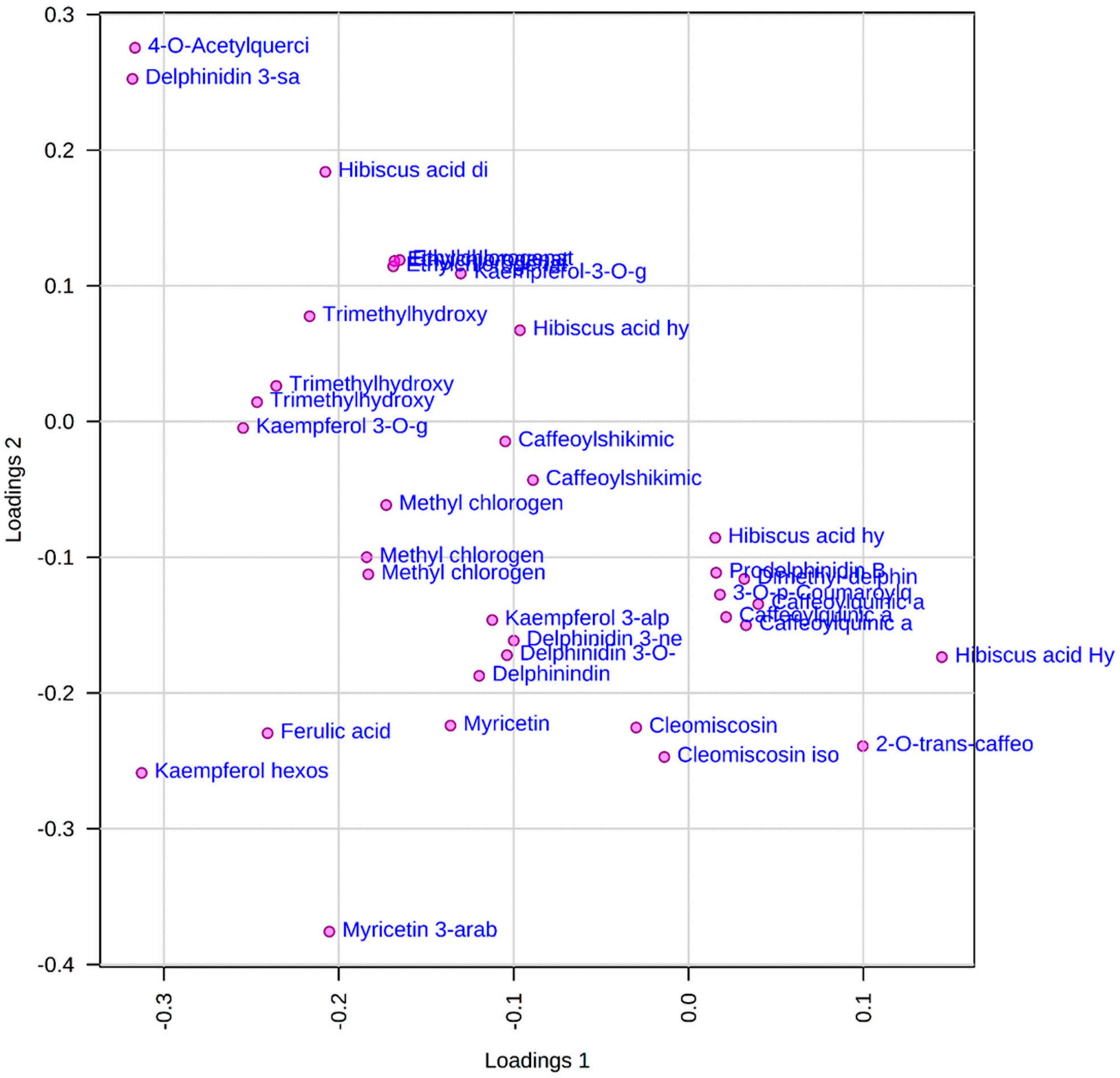

Figure S8. Principal component analysis (PCA) loading plot of metabolites identified from Hibiscus calyces using different extraction methods with different proportions of methanol/water.

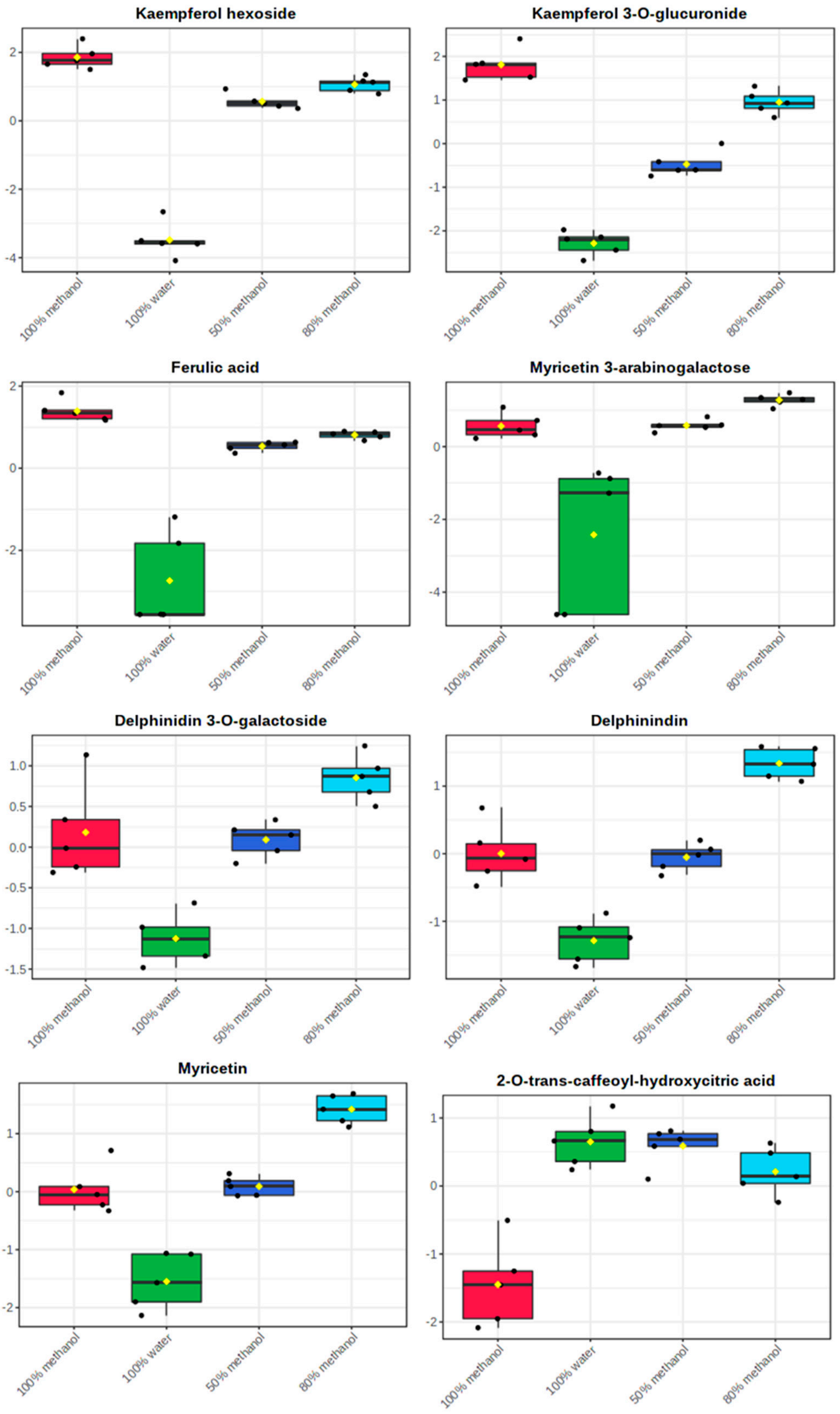

Figure S9. Metabolites with varied solubility in methanol. The y-axis represents the log2 value of metabolite abundance.

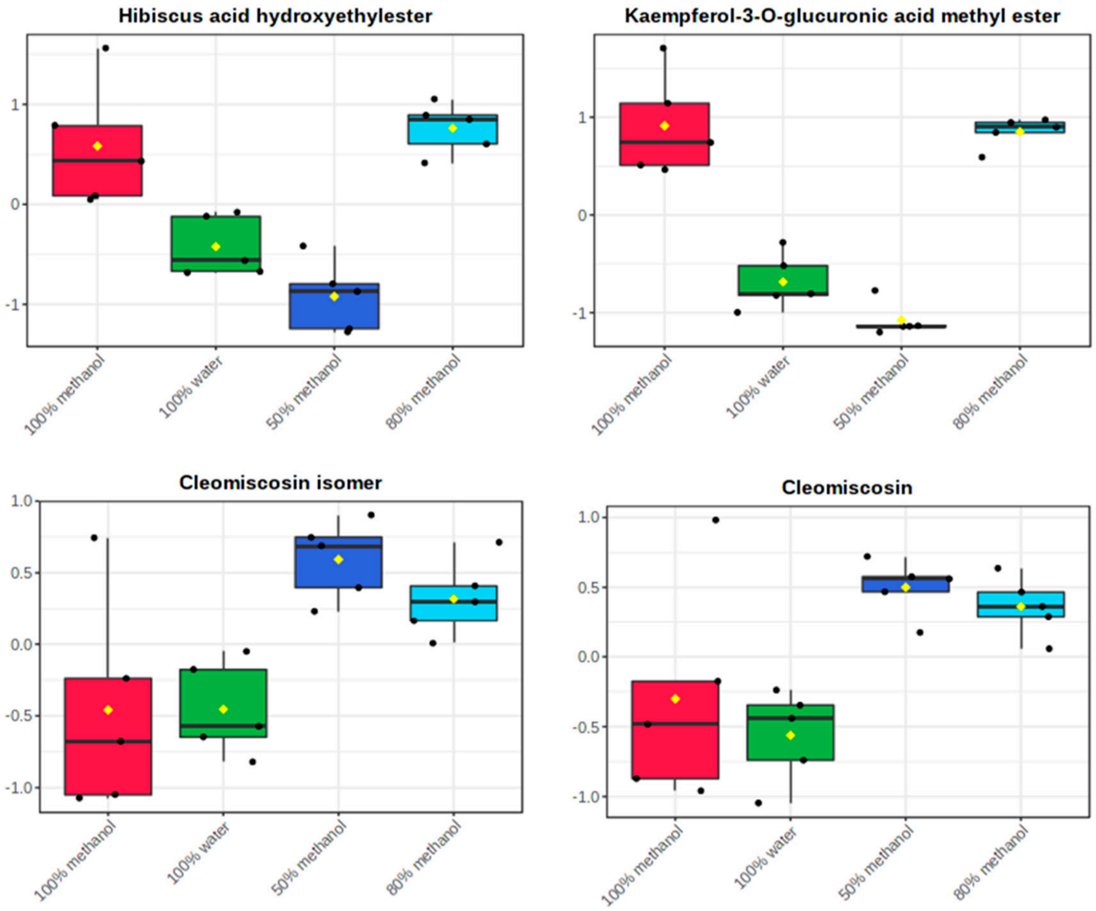

Figure S10. Metabolites with showed highest or lowest solubility in 50% methanol. The y-axis represents the log2 value of metabolite abundance.
